# *Listeria monocytogenes* Assessment in a Ready-to-Eat Salad Shelf-Life Study Using Conventional Culture-Based Methods, Genetic Profiling, and Propidium Monoazide Quantitative PCR

**DOI:** 10.3390/foods10020235

**Published:** 2021-01-24

**Authors:** Rita Bernardo, Ana Duarte, Luís Tavares, António Salvador Barreto, Ana Rita Henriques

**Affiliations:** CIISA–Centre for Interdisciplinary Research in Animal Health, Faculty of Veterinary Medicine, University of Lisbon, 1300-477 Lisbon, Portugal; ritadcbernardo@gmail.com (R.B.); anaduarte@fmv.ulisboa.pt (A.D.); ltavares@fmv.ulisboa.pt (L.T.); asbarreto@fmv.ulisboa.pt (A.S.B.)

**Keywords:** *Listeria monocytogenes*, ready-to-eat food, shelf-life, culture-based methods, propidium monoazide, quantitative polymerase chain reaction

## Abstract

Listeriosis is almost entirely transmitted through foods contaminated with *Listeria monocytogenes*. Ready-to-eat foods present a particular challenge due to their long refrigerated shelf-life, not requiring any heat treatment before consumption. In this work, a shelf-life assessment of an industrially produced ready-to-eat salad was performed using conventional culture-based and molecular methods. *L. monocytogenes* isolates were confirmed and serogrouped using multiplex PCR, and genetic subtyping was performed by pulsed-field gel electrophoresis (PFGE). PMAxx-qPCR was used as an alternative method for *L. monocytogenes* quantification in foods. Salad samples were kept at 4 °C, 12 °C, and 16 °C for eight days and analysed. At 4 °C, acceptable results were obtained considering hygiene indicators, i.e., Enterobacteriaceae (ranging from 3.55 ± 0.15 log cfu/g to 5.39 ± 0.21 log cfu/g) and aerobic mesophilic colony counts (5.91 ± 0.90 log cfu/g to 9.41 ± 0.58 log cfu/g) throughout the study, but the same did not happen at 12 °C and 16 °C. *L. monocytogenes* culture-based quantification exhibited low numbers (<1 log cfu/g) for all temperatures. From 30 presumptive isolates, 10 (33.3%) were confirmed as *L. monocytogenes* with the majority belonging to serogroup IVb. PFGE subtyping showed that 7 of the 10 *L. monocytogenes* isolates had 100% of pulsotype similarity, suggesting a possible common contamination source. PMAxx-qPCR revealed a statistically higher *L. monocytogenes* quantification (>3 log cfu/g) when compared to the conventional culture-based method, suggesting viable but non-culturable forms. Taken together, results underline the need to combine conventional methods with more sensitive, specific, and rapid ones for *L. monocytogenes* assessment in ready-to-eat foods shelf-life studies to reduce the potential risk for consumers.

## 1. Introduction

Contemporary lifestyles have a major influence on food consumption patterns, and one of the major trends is the growing preference for convenience foods to which ready-to-eat (RTE) salads are well associated [1,2]. Prepacked RTE salads include several ingredients, typically containing raw cuts of vegetables and other cooked and smoked ingredients [3]. RTE salads endure extensive handling processes during preparation, being exposed to several contamination opportunities, including recontamination from processing surfaces and equipment, following a listericidal treatment [4,5]. RTE salads have long refrigerated shelf-lives that allow the multiplication of psychrotrophic *Listeria monocytogenes* and do not require a heating step before consumption. These foods should be regarded as potential vehicles of transmission of human listeriosis [6,7]. Listeriosis is a rare severe human infection, with high fatality rates, and is almost exclusively transmitted by food consumption [8,9]. In the last decade, European member states have reported thousands of confirmed human listeriosis cases per year, with high fatality rates [8]. This trend has also been reported by the Centers for Disease Control and Prevention (CDC) in the United States of America [9]. European legislation considers the limit of 100 colony-forming units of *L. monocytogenes* per gram (cfu/g) of RTE food during shelf-life [10]. The International Organization for Standard method for *L. monocytogenes* enumeration (ISO 11290-2) is considered the reference method in the quantitative criteria of European Commission Regulation No. 2073/2005 [10]. However, food products are usually contaminated at low levels, and if *L. monocytogenes* is present below the ISO 11290-2 lower limit of detection (10 cfu/g), the method lacks sufficient sensitivity to reliably quantify it [11]. Moreover, in food, *L. monocytogenes* is often affected by a variety of processing hurdles, including heating, freezing, and exposure to disinfectants. This may lead to the loss of their ability to grow on selective media-cultivability, while viability remains unaltered, becoming viable but non-culturable (VBNC) bacteria [11,12].

When addressing food shelf-life, especially foods with a short shelf-life, the rapid delivery of results is essential. Several authors have addressed possible alternatives to conventional culture-based methods, and polymerase chain reaction (PCR) and real-time quantitative PCR (qPCR) have been tested for this purpose [13,14,15,16,17]. The development of more sensitive, rapid, and specific methods than plate counts for the quantification of viable and VBNC *L. monocytogenes* is fundamental to allow the extension of the commercial life of short-term food products [18,19,20].

Molecular methods present considerable advantages in food microbiological safety assessment, but one of the main flaws of DNA-based methods is their inability to distinguish viable and dead cells because the DNA of a dead cell can persist in the food matrix, leading to an overestimation of target bacteria concentrations. This particular issue has greatly limited the application of molecular-based methods in food microbiology routine monitoring because non-viable cells are frequently present after food processing [21,22]. The use of propidium monoazide (PMA) prior to DNA extraction allowed the discrimination of viable and dead cells [20,23,24]. PMA action is based on the presence of an azide group that allows cross-linking of the dye to the DNA of dead cells with compromised membrane integrity. Because the dye is cell-membrane impermeable, it can be used to selectively and permanently modify the DNA from dead cells with compromised membrane integrity while leaving the DNA from viable cells intact. The induced DNA modification will inhibit amplification in subsequent PCR reactions, while the DNA of viable cells, which is protected by intact membranes, will be detected by qPCR. [22,24,25]. The use of PMA has been successfully integrated with qPCR assays for the differentiation of viable and dead *L. monocytogenes* cells in different food samples [18,26]. On the other hand, for source tracking and epidemiologic investigation of *L. monocytogenes*, pulsed-field gel electrophoresis (PFGE) is a very useful tool for subtyping because of its high reproducibility, robustness, and discriminating power [27,28,29].

In this work, a shelf-life assessment of a ready-to-eat salad produced in an industry with a history of contamination by *L. monocytogenes* was performed. Together with conventional culture-based methods, molecular-based approaches were used to assess *L. monocytogenes* detection and quantification. For this purpose, the recovered isolates were confirmed and serogrouped using a multiplex PCR, and genetic subtyping was performed using PFGE, aiming to determine strains relatedness. PMAxx-qPCR was also used to address rapid and alternative methods of *L. monocytogenes* quantification in foods.

## 2. Materials and Methods

### 2.1. Ready-to-Eat (RTE) Salad Production Process

The studied ready-to-eat (RTE) salad was produced in an officially approved food-producing industry, located in an industrial park in Lisbon. The salad’s ingredients include disinfected green cuts (lettuce, radicchio, and endives in variable proportions), oven-grilled diced chicken breast, cured grated cheese, and fried bacon pieces, acquired as refrigerated pre-packaged ingredients. The salad is manually assembled in a temperature-controlled (10–12 °C) room, packaged in a polyethylene terephthalate lidded salad bowl with environmental atmosphere, immediately sealed, and stored at 5 °C during its six-day commercial shelf-life.

### 2.2. Ready-to-Eat Salad Sampling and Storage during Shelf-Life Testing

Twenty-seven salad samples were randomly collected from nine different batches produced in different weeks over a period of three months and transported to the laboratory in less than 2 h using an isothermal box. Salad samples were incubated at 4 °C, 12 °C, and 16 °C, and were analysed at day 0, day 4, and day 8. Three independent replicates (different batches) were performed for each temperature.

### 2.3. Physicochemical Analyses

#### 2.3.1. Water Activity (*a*_w_) Determination

For *a*_w_ determination EN ISO 21807:2004 [30] standard was followed using a water activity meter with AW-40 probe (HygroLab C, Rotronic Instruments, West Sussex, UK), maintained at 25 °C ± 2 °C. For this purpose, three independent measurements were performed in each sample after homogenisation, in the considered sampling time points.

#### 2.3.2. Potential of Hydrogen (pH) Determination

For pH determination, three independent measurements were performed in each sample after homogenisation, in the considered sampling time points. The evaluation was conducted according to NP-3441 (1990) [31], using a potentiometer (HI 99163, Hanna Instruments, Woonsocket, RI, USA).

### 2.4. Microbiological Analyses

For microbiological analysis purposes, salad samples were prepared according to ISO 6887-2:2003 [32]. Enterobacteriaceae colony counts were carried out according to ISO 21528-2:2017 [33], and aerobic mesophilic colony enumeration was performed conforming to ISO 4833-1:2013 [34]. *L. monocytogenes* detection and enumeration were performed according to ISO 11290-1 [35] and 2:2017 [36], respectively. Salad extracts were kept for further molecular assessment purposes and comprised the initial suspension of ISO 11290-2—the salad’s test portion and the diluent, i.e., buffered peptone water (BPW; Scharlab, S.L., Barcelona, Spain). All countings were expressed as log colony-forming units per gram of salad (cfu/g).

### 2.5. L. monocytogenes Confirmation and Serogrouping

Presumptive colonies of *L. monocytogenes* were assessed using Kérouanton et al. (2010) [37] protocol that combines a multiplex PCR assay with an additional PCR for the amplification of *fla*A gene, enabling *Listeria* genus and *L. monocytogenes* species-specific recognition, in addition to serogroups identification.

### 2.6. Pulsed-Field Gel Electrophoresis Typing

*L. monocytogenes* confirmed isolates were tested by pulsed-field gel electrophoresis (PFGE), following PulseNet standardised protocol for *L. monocytogenes* typing [38]. In brief, genomic DNA in 1% agarose (SeaKem Gold Agarose, Cambrex, East Rutherford, NJ, USA) plugs was digested in separate reactions with 10 U/μL of *Asc*I (New England Biolabs, Ipswich, MA, USA) for 4 h at 37 °C, and with 50 U/μL of *Apa*I (New England Biolabs, Ipswich, MA, USA) for 4 h at 25 °C. Electrophoresis was performed over 19 h at 14 °C in 0.5× Tris-borate-EDTA buffer (NZYTech, Lisbon, Portugal) using 1% SeaKem Gold Agarose gels, with the following conditions: 6 V/cm, initial pulsed time of 4.0 s, final pulsed time of 40 s, and included angle of 120° in a CHEF-Dr. III System apparatus (Bio-Rad Laboratories, Hercules, CA, USA). Afterwards, ethidium bromide (Sigma, St. Louis, MO, USA) was used to stain the gels that were photographed under UV transillumination.

### 2.7. PMAxx-qPCR Assay

#### 2.7.1. Bacterial Strains Preparation

To validate PMAxx-qPCR specificity, a target strain (*L. monocytogenes* CECT 935) and a non-target strain (*Escherichia coli* DSMZ 682) were used. Briefly, both strains were cultivated according to their specific requirements and submitted to the PMAxx treatment and genomic DNA extraction prior to qPCR.

#### 2.7.2. PMAxx Treatment and Genomic DNA Extraction

For propidium monoazide (PMAxx) (Biotium Inc., Hayward, CA, USA) treatment, samples were treated according to Zhang et al. (2014) [39] with few modifications. Using transparent microtubes, PMAxx™ (20 mM stock in H_2_O) was added to the salad extracts for a final concentration of 80 µM, incubating for 5 min in darkness, at room temperature, and shaken with an Orbit™ P4 Digital Shaker (Labnet Int., Edison, NJ, USA) at 40 rpm to promote selective penetration of PMAxx into dead cells. Microtubes were then horizontally laid on crushed ice, using an Orbit™ P4 Digital Shaker with brief shaking, and exposed to a 1000-W halogen light source (Osram Licht AG, Munich, Germany) for 15 min, to cross-link PMAxx with the DNA and convert unintercalated PMAxx to hydroxylamino propidium [40]. The light source was positioned to be at approximately 40 cm from the microtubes to avoid excessive heating. After the photo-induced cross-linking, bacteria were harvested by centrifugation at 5000 rpm for 10 min. To check for *L. monocytogenes* isolates viability, the pellet was suspended in BPW (Scharlab, S.L., Barcelona, Spain) and plated in Agar *Listeria* Ottaviani & Agosti ALOA medium (BioMérieux, Marcy l’Etoile, France). Additionally, as a control, salad extracts samples were boiled (100 °C for 1 h) in a laboratory heat-block, submitted to the PMAxx treatment as previously described, and further tested.

Genomic DNA extraction from PMAxx treated samples was performed using the guanidine thiocyanate (GES) method [41]. The internal extraction control DNA of the Genesig^®^ advanced kit *Listeria monocytogenes* invasion-associated Protein p60 (iap) gene (PrimerDesign™ Ltd., Rownhams, UK) was included in the GES reagent addition step. The resulting DNA was stored at 4 °C for further assessment.

2.7.3. qPCR Assay

*L. monocytogenes* quantification was performed using Genesig^®^ advanced kit *Listeria monocytogenes* invasion-associated Protein p60 (iap) gene (PrimerDesign™ Ltd., Rownhams, UK) with primers directed to amplify the *iap* gene (Table 1). According to the manufacturer, the kit includes specific and exclusive primers and probe sequences for in vitro *L. monocytogenes* quantification, having 100% homology with over 95% of reference sequences in National Center for Biotechnology Information Database and analytical sensitivity of <1 × 10^2^ target copies.

Each PCR reaction incorporated 5 μL of template DNAs, 10 μL of PrecisionPLUS 2X qPCR MasterMix (PrimerDesign™, Ltd.), 1 μL of *L. monocytogenes*-specific primer/probe mix (detected through the FAM channel), 1 μL of internal extraction control primer/probe mix (detected through the VIC channel) and 3 μL of nuclease-free water. The assay was conducted using ABI StepOnePlus™ Real-Time PCR System (Applied Biosystems, Foster City, CA, USA) under the following cycling conditions: 2 min at 95 °C; 50 cycles of 10 s at 95 °C; and 60 s at 60 °C.

Genomic DNA from *L. monocytogenes* CECT 935 served as a positive control for the reaction. Negative controls using nuclease-free PCR grade water without template control, and *Escherichia coli* DSMZ 682 genomic DNA were included in each run.

To examine the PMAxx-qPCR sensitivity, serial dilutions of target strain *L. monocytogenes* CECT 935 at known concentrations were used, ranging from 10^1^–10^6^ cfu/mL. The suspensions were treated with PMAxx, followed by genomic DNA extraction, PMAxx-qPCR detection assay, and a standard curve was generated.

Three experimental trials were carried out on different days, and three replicates were analysed each time for each condition.

### 2.8. Statistical Analyses

Results from microbiological and physicochemical assays were analysed by calculating the average and standard deviation of replicates, corresponding to three batches, for the three different temperatures, using GraphPad Prism 5 (GraphPad Software, La Jolla, CA, USA). To compare results obtained at the three assessed temperatures, one-way analysis of variance (ANOVA) was used, followed by Tukey’s multiple comparison test, and *p* values of 0.05 or less were considered significant.

To compare the two *L. monocytogenes* quantification methods, i.e., viable cell counts using the culture-based method and PMAxx-qPCR, a *t*-test for paired samples was performed for each temperature (4 °C, 12 °C, and 16 °C), using GraphPad Prism 5 (GraphPad Software, La Jolla, CA, USA).

A dendrogram based on *L. monocytogenes* pulsotypes was created using BioNumerics software v6.10 (Applied Maths, Sint-Martens-Latem, Belgium). To determine strains relatedness, an optimisation setting and a band-position tolerance of 1.5% were used to analyse *L. monocytogenes* PFGE patterns with *Asc*I and *Apa*I restriction. The unweighted pair group method with arithmetic averages and band-based Dice correlation coefficient were considered for cluster analysis purposes.

## 3. Results

### 3.1. Physicochemical Analyses—a_w_ and pH Measurements

The *a*_w_ and pH values measured in RTE salad samples at 4 °C, 12 °C, and 16 °C throughout the eight days of study are presented in Table 2. The obtained results confirmed that the studied salad is a food able to support *L. monocytogenes* growth, according to Regulation (EC) No. 2073/2005 of 15 November 2005, amendments on microbiological criteria for foodstuffs, and the European Reference Laboratory Technical guidance for shelf-life studies on *L. monocytogenes* in foods [10,43].

In all samples and temperatures, *a*_w_ results did not reach the lower and upper *L. monocytogenes* growth limits, which are 0.93 and >0.99, respectively [43].

Considering pH values, while no differences were detected at 4 °C (*p* > 0.05), significant differences (*p* < 0.05) were found at 12 °C and 16 °C throughout the assessed storage period of eight days, which could be due to an overgrowth of raw ingredients microbiota [5]. Still, pH values did not reach *L. monocytogenes* lower (pH = 4.2) and upper growth limit (pH = 9.5) considering all samples and temperatures [43].

### 3.2. Microbiological Analyses

#### 3.2.1. Hygiene Indicators

**Figure 1:**
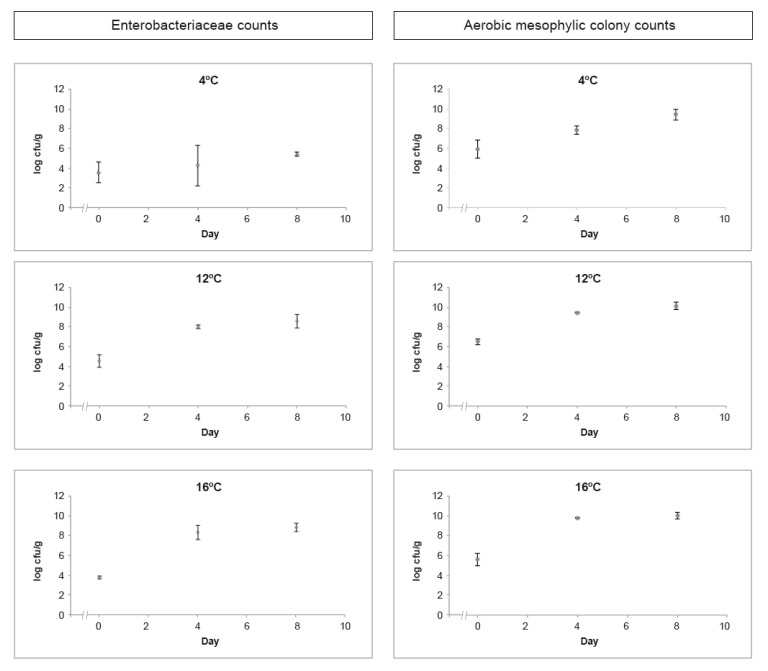
Average and standard deviation of the assessed hygiene indicators (Enterobacteriaceae and aerobic mesophilic colony counts) in log cfu/g obtained from RTE salads at 4 °C, 12 °C, and 16 °C throughout the shelf-life study.

Enterobacteriaceae and aerobic mesophilic colony enumeration results through the eight days of study, considering the assessed temperatures of 4 °C, 12 °C, and 16 °C, are presented in Figure 1.

Enterobacteriaceae countings in RTE salads stored at 4 °C revealed values ranging from 3.55 ± 0.15 log cfu/g to 5.39 ± 0.21 log cfu/g during the studied eight days, at 12 °C values ranged from 4.23 ± 0.57 log cfu/g to 7.95 ± 0.63 log cfu/g, while at 16 °C values ranged from 3.79 ± 0.15 log cfu/g to 8.80 ± 0.43 log cfu/g (Figure 1). These findings are in agreement with Manios et al. (2013) [44] that reported an increase in Enterobacteriaceae countings in salads stored for 10 to 12 days at 8 °C. Significant differences (*p* < 0.05) were detected on Enterobacteriaceae countings on the first and last day of the challenge test, at 12 °C and 16 °C, indicating the presence of psychrotrophic Enterobacteriaceae that are able to multiply in chilled food. The Enterobacteriaceae family is commonly used to assess the adequacy of food processing and hygiene practices [45]. Enterobacteriaceae counts higher than 4 log cfu/g in RTE foods would be unsatisfactory in terms of hygiene [46]. However, high countings could be expected in raw salads and vegetables, ranging from 4 log cfu/g to 8 log cfu/g, since some members of this family are natural colonisers of fresh vegetables [47]. Therefore, the high values of Enterobacteriaceae observed in the studied RTE salads, with fresh vegetables as ingredients, may not indicate a lack of hygiene practices because the use of sanitising rinses may reduce but not entirely remove these organisms [46,48].

Aerobic mesophilic colony counts in RTE salads at 4 °C revealed values ranging from 5.91 ± 0.90 log cfu/g to 9.41 ± 0.58 log cfu/g during eight days of incubation. At 12 °C, the values ranged from 6.47 ± 0.31 log cfu/g to 10.06 ± 0.37 log cfu/g, and at 16 °C the values ranged from 5.63 ± 0.90 log cfu/g to 10.01 ± 0.35 log cfu/g (Figure 1). These findings are in line with the ones reported by Omac et al. (2018) [49], assessing the growth of total aerobic microorganisms on fresh spinach leaves at 3 °C, 5 °C, and 8 °C during 16 days of storage. Skalina and Nikolajeva (2010) [50] also found a significant increase in total aerobic microorganisms on RTE mixed salads during 48 h of storage at 3 °C and 7 °C. Aerobic mesophilic microorganisms’ enumeration provides useful information as a quality indicator in food shelf-life testing but cannot contribute directly towards a safety assessment of RTE foods [46,48]. There are many factors contributing to the rate of microbial growth, including the type of food product and its processing, the type of packaging, and shelf-life storage temperature [46,51]. For raw RTE foods such as salads, aerobic mesophilic colony counts are likely to be higher, between 10^6^ and 10^8^ cfu/g, limiting these RTE foods’ shelf-life because spoilage may occur rapidly and visibly [46]. When stored at 4 °C, the assessed RTE salad presented acceptable results considering hygiene indicators and overall visual quality throughout the eight days of study. Nevertheless, as expected, the same did not happen when stored at 12 °C and 16 °C, revealing the inadequacy of those temperatures to store these salads and the relevance of strict temperature control from processing to consumption.

#### 3.2.2. Detection and Enumeration of *L. monocytogenes*, Confirmation, and Serogrouping

Throughout the study, it was possible to detect presumptive colonies of *L. monocytogenes* (*n* = 30) in nine samples of RTE salad, with all countings < 1 log cfu/g (Table 3), sustaining the ability of *L. monocytogenes* to develop in the assessed RTE salad. Although time and temperature abuse during shelf-life may influence the level of *L. monocytogenes* in these salads, no significant differences were found between incubation temperatures results. Moravkova et al. (2017) [52] reported similar findings when quantifying *L. monocytogenes* in ready-to-eat vegetables using culture-based methods (ISO 11290-2).

All *L. monocytogenes* presumptive isolates (*n* = 30) were tested, being identified as *Listeria* spp., but only 10 (33.3%) were confirmed as *L. monocytogenes* (Table 3). Because ISO 11290-2:2017 highlights that some strains of *L. monocytogenes* can exhibit a very weak halo, or even no halo, colony’s collection was performed according to these instructions [36]. Moreover, ISO 11290-2:2017 also mentions that *L. ivanovii* colonies may have the same morphological aspect as *L. monocytogenes,* i.e., blue-green colonies with an opaque halo [36]. These assumptions might explain the confirmation of only 10 isolates as *L. monocytogenes* by PCR. Still, these results were not surprising because the industrial unit had a history of *L. monocytogenes* occurrence in final products. The presence of other *Listeria* species, in addition to *L. monocytogenes*, might be used as a hygiene indicator, and preventive and corrective actions could be considered [53,54].

Taking together the obtained results for hygiene indicators and *L. monocytogenes* using conventional culture-based methods, when stored at 4 °C, this RTE salad could have an attributed shelf-life of eight days, complying with Regulation (EC) No. 2073/2005 food safety criteria.

Among the confirmed *L. monocytogenes* isolates, three molecular serogroups were identified—IIa, IIb, and IVb (Table 4).

The majority of isolates belonged to serogroup IVb (80%), which is also commonly associated with human infection [55]. Moreover, the number of reported cases of listeriosis associated with serogroup IVb appears to be increasing [8]. In the European Union, *L. monocytogenes* was detected in 1.5% of 2583 RTE salads sampled in 2018, using culture-based methods; considering all RTE food categories, serogroup IVb was the most common [8]. Other studies have reported similar findings. In Poland, 0.7% of 20,304 samples of RTE meat-based foods were positive for *L. monocytogenes* and 105 isolates were obtained, with the majority belonging to serogroup IVb (31.4%), followed by serogroups IIb (24.8%), IIa (21.9%), and IIc (2.9%) [56]. In Morocco, from 1096 analysed RTE food samples, 1.5% were positive for *L. monocytogenes* with a predominance of serogroup IVb isolates (87.5%), followed by IIa (12.5%) [57]. The presence of serogroup IVb among RTE foods indicates a potential public health risk due to their higher pathogenic potential for consumers. Serogroup IIa strains are believed to be better adapted to survive and multiply in the environment, being common in foods and food related-environment [58,59]. In a work aiming to determine *L. monocytogenes* occurrence and diversity in a meat-processing facility, from 268 environmental and food samples, 70 were found to be positive [60]. The isolates were assigned into four serogroups, with the majority (44.1%) belonging to serogroup IIa, followed by IIb (28.6%), IIc (19.5%), and IVb (7.8%) [60]. In another study in delicatessen producing industries, food and environment samples (*n* = 80) were collected, of which 14 were positive for *L. monocytogenes,* and 62 isolates were obtained. More than one *L. monocytogenes* serogroup was identified in some of the samples, and serogroups IIb and IIa were the most common [61].

#### 3.2.3. PFGE Typing

**Figure 2:**
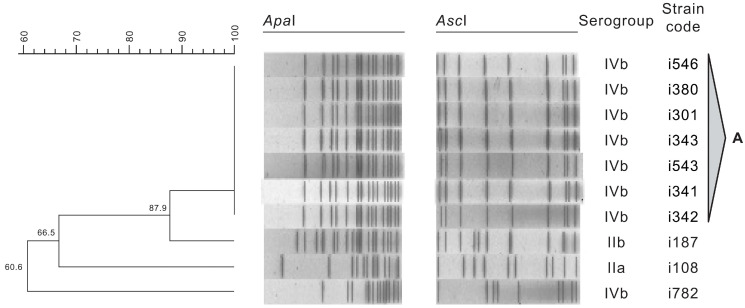
Dendrogram of *L. monocytogenes* PFGE profiles and serogroups.

The resulting dendrogram obtained from the analysis of the restriction profiles of *L. monocytogenes* isolates with *Apa*I and *Asc*I is shown in Figure 2, along with the serogroups. The 10 *L. monocytogenes* isolates from different RTE salad batches presented four PFGE types. Pulsotypes were considered to be clones when presenting 95% or more of similarity.

Pulsotype A included the majority of the assessed isolates (70%), all belonging to serogroup IVb. These strains were recovered from two different RTE salad batches (produced with 42 days of interval), suggesting a common source of contamination, because all the isolates in this pulsotype shared 100% of similarity. It is important to highlight that these strains belonged to serogroup IVb, which is commonly associated to human disease, occurring in a RTE salad that will not undergo any heat-treatment prior to consumption [55,62]. Contrastingly, isolates i187, i108, and i782, belonging to serogroups IIb, IIa, and IVb respectively, displayed distinct profiles. Yu and Jiang (2014) [63] also found distinct profiles in approximately 30% of the studied PFGE profiles when assessing *L. monocytogenes* isolates collected from retailed foods in Henan, China. The occurrence and persistence of *L. monocytogenes* in food processing premises and surfaces are important factors for the transmission of this opportunistic pathogen to food [52]. A thorough sampling plan should be considered during a prolonged time frame, in order to conclude on the contamination routes and eventual persistence of *L. monocytogenes* strains in the assessed food industry, and in its suppliers premises, including final products, raw materials, and food-related environment samples.

#### 3.2.4. PMAxx-qPCR Assay

**Figure 3:**
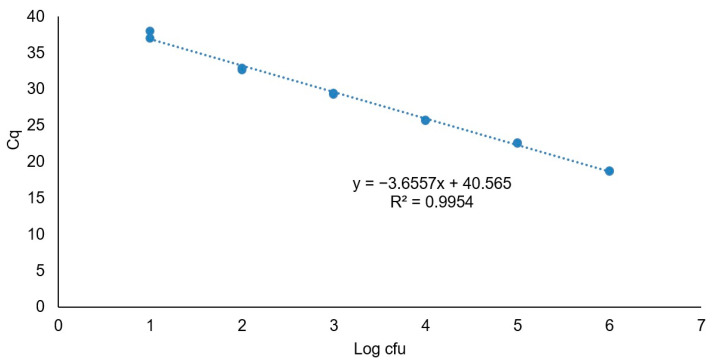
Standard curve of the 124 bp *iap* gene fragment of *L. monocytogenes* CECT 935 obtained with quantification cycle (Cq) plotted against the logarithmic concentration of the serial dilutions. The trend line equation and corresponding square regression coefficient (*R*^2^) are shown.

The Cq values obtained by PMAxx-qPCR were quantified using a relative standard curve generated with the positive control DNA at known concentrations (Figure 3). The standard curve exhibited a linear relationship with a curve slope of −3.66 and a correlation coefficient (*R*^2^) of 0.99. Based on these data, the assay had an efficiency value (*E*) of 92%.

**Figure 4:**
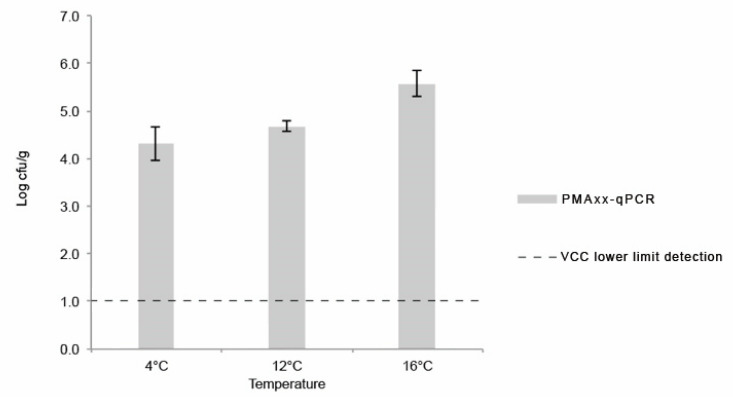
Average and standard-deviation of *L. monocytogenes* concentration (log cfu/g) obtained by PMAxx-qPCR on the final day of each assay (day 8). The dotted line represents uncertainty of viable cell countings method.

The obtained concentrations of *L. monocytogenes* on the last day (day 8) of the shelf-life assay using PMAxx-qPCR technique are shown in Figure 4. For every assay, the concentration of *L. monocytogenes* (log cfu/g) obtained by PMAxx-qPCR was significantly higher (*p* < 0.05) than the one obtained by colony-counts in ALOA using ISO 11290-2:2017 quantification method (Table 3). At least a 3 log cfu/g difference was obtained, considering the lower limit of quantification of the conventional method (ISO 11290-2:2017). Similar results were reported by other authors when comparing both methods for bacterial quantification in food and food-processing environments [22,64,65]. No amplification was detected in salad extracts controls that were submitted to boiling prior to PMAxx-qPCR.

While the RTE salad was compliant with food safety criteria for RTE foods able to support the growth of *L. monocytogenes* during shelf-life when considering culture-based methods (<1 log cfu/g), the same did not happen when using PMAxx-qPCR results, which were above the food safety criteria of 2 log cfu/g [10]. The underestimation obtained using the conventional culture-based method suggests the occurrence of viable but non-culturable (VBNC) *L. monocytogenes* in the food samples, and while qPCR is able to detect VBNC bacteria, traditional methods lack the sensibility to do so. In line with these results, Barreta et al. (2019) [66] quantified *L. monocytogenes* by culture-based methods and PMA-qPCR, and while it was not possible to quantify it using culture-based methods, PMA-qPCR yielded levels that suggested a VBNC state [66]. In food, *L. monocytogenes* is often affected by processing treatments that may hamper bacterial cultivability, while viability remains unaltered [11,12]. PMAxx has the ability to penetrate cells with compromised membrane integrity, covalently binding to DNA and free DNA, preventing PCR amplification and presenting a higher discriminative effect when compared to PMA in complex matrixes [65]. However, Nocker and Camper (2009) [67] highlighted the potential risk of overestimating VBNC cells because dead cells may present an intact membrane. In this way, it is necessary to be cautious and critical when using PMAxx-qPCR for *L. monocytogenes* quantification in terms of food safety [11,68].

Simultaneously, when solely using traditional enumeration methods, false negative results might be expected, hampering the implementation of mitigation measures by the producing industry and risking the presence in the market of food unacceptably contaminated with *L. monocytogenes* [18,20,69]. Therefore, PMAxx-qPCR should not be disconnected from other classical techniques, but rather considered a complementary tool, because it is a powerful approach to access bacterial viability, allowing for an easier, sensitive, specific, and time-saving *L. monocytogenes* quantification. In the future, this promising technique may become a reliable and accurate method to be transferred from expert research to routine laboratories in the food industry [11,20]. This is especially important when considering RTE foods due to their short commercial shelf-life [64,70].

## 4. Conclusions

The obtained results of *a*_w_ and pH confirmed that this study’s RTE salad was able to support *L. monocytogenes* growth. Hygiene indicators assessment revealed that both Enterobacteriaceae and aerobic mesophilic colonies reached high numbers at 12 °C and 16 °C through the eight days of study, reinforcing the need to keep these RTE salads at consistently low temperatures during all of the commercial shelf-life. *L. monocytogenes* quantification by culture-based methods consistently displayed contamination levels below the detection limit of the method throughout the study. Considering the obtained results using conventional culture-based methods, a shelf-life of eight days could be attributed to the studied RTE salad when kept at 4 °C.

Using multiplex PCR, all of the 30 presumptive isolates were confirmed as *Listeria* spp., but only 10 were *L. monocytogenes*, which were assigned to serogroups IVb, IIa, and IIb. PFGE results revealed that 7 of the 10 *L. monocytogenes* isolates shared the same pulsotype (100% of similarity), suggesting a possible common source.

Considering *L. monocytogenes* quantification, discrepant results were obtained when comparing culture-based methods and PMAxx-qPCR, with the former not being able to reflect the same level of contamination as the latter, suggesting the occurrence of VBNC *L. monocytogenes*.

To establish the shelf-life of food products, the producing unit is required by law to use conventional culture-based methods. Still, after leaving the producer, food products are transported, stored, and handled, enduring varying temperatures that affect microbial growth, and simultaneously, the food product’s shelf-life. This study’s results underline the need to combine conventional methods with more sensitive, specific, and rapid methods for *L. monocytogenes* quantification, especially when addressing RTE foods that will not have a listericidal treatment before consumption, in order to mitigate the potential risk for consumers.

## Figures and Tables

**Table 1:** Specific amplicon context sequence used in the quantitative PCR (qPCR) assay (PrimerDesign™ Ltd., Rownhams, UK). For proprietary reasons, the primer/probe sequences cannot be disclosed, but the details provided are compliant with the minimum information for publication of quantitative real-time PCR experiments according to Bustin et al. (2011) [42].

Gene	Accession Number	Anchor Nucleotide	Amplicon Length (bp)
*iap*	AF500174.1	285	124

**Table 2:** Average and standard deviation for *a*_w_ and pH values obtained in ready-to-eat (RTE) salads at 4 °C, 12 °C, and 16 °C throughout the eight days of study.

Storage Temperature		Day 0	Day 4	Day 8
***a*** **_w_**	4 °C	0.955 ± 0.001	0.957 ± 0.006	0.962 ± 0.009
	12 °C	0.966 ± 0.001	0.957 ± 0.002	0.971 ± 0.008
	16 °C	0.980 ± 0.002	0.958 ± 0.001	0.963 ± 0.007
**pH**	4 °C	5.930 ± 0.033	6.113 ± 0.191	6.251 ± 0.561
	12 °C	6.021 ± 0.080	6.427 ± 0.400	6.632 ± 0.554
	16 °C	6.647 ± 0.491	7.309 ± 0.325	7.625 ± 0.167

**Table 3:** *L. monocytogenes* presumptive colonies (*n* = 30) collected in salad samples throughout the shelf-life study using conventional enumeration method (ISO 11290-2:2017). *L. monocytogenes* confirmed isolates (*n* = 10) using multiplex PCR are written in bold.

Salad Code	Incubation Temperature	Presumptive Colonies (*n*) Collected throughout Shelf-Life								
		Day 0			Day 4			Day 8		
		A ^a^	B ^a^	C ^a^	A ^a^	B ^a^	C ^a^	A ^a^	B ^a^	C ^a^
1	4 °C	0	0	1	0	0	0	0	0	1
2	4 °C	0	0	0	0	0	0	1	1	0
3	4 °C	1	1	0	3	1	0	0	0	0
4	12 °C	0	0	0	0	0	0	1	1	0
5	12 °C	0	0	0	1	1	0	1	1	0
6	12 °C	0	0	1	0	0	0	0	0	0
7	16 °C	0	0	0	1	1	0	1	2	0
8	16 °C	0	0	0	1	1	0	2	0	0
9	16 °C	0	0	0	2	0	0	1	1	0

^a^—different capital letters represent experimental replicates (different salad production batches).

**Table 4:** Description of the obtained serogroups among *L. monocytogenes* confirmed isolates (*n* = 10).

Serogroup	Proportion	Isolate Code
IIa	1 (10%)	i108
IIb	1 (10%)	i187
IVb	8 (80%)	i380, i301, i341, i342, i343, i543, i546, i782
